# The impact of price and image warnings on the social perception of gifting cigarettes in China

**DOI:** 10.18332/tid/176806

**Published:** 2024-01-24

**Authors:** Guang Xu, Yibin Shi, Kecheng Du, Gang Wang, Liyun Wu

**Affiliations:** 1 School of Journalism and Communication, Wuhan University, Wuhan, China; 2 School of Social Work, Norfolk State University, Norfolk, United States

**Keywords:** social value, cigarette price, image warning label, survey experiment

## Abstract

**INTRODUCTION:**

The tobacco gift-giving culture in China poses a significant challenge to public health; however, there is limited research on effectively curbing the tobacco gift-giving culture and its associated tobacco gift consumption. This study examines the potential impact of two tobacco control measures that the Chinese government may consider adopting on cigarette gifting behavior in the future in Chinese society.

**METHODS:**

This study employed a randomized survey experiment to examine the effects of cigarette price treatment and pictorial health warning labels (HWLs) on cigarette gifting. The total sample size of this study is 1035. Four groups of participants were presented with representative cigarettes categorized into high-, medium-, and low-priced products, along with different prices (normal and double) or external packaging imagery (normal and pictorial HWL versions).

**RESULTS:**

The price of cigarettes for personal consumption forms an L-shaped distribution, and the price of cigarette gifts forms a W-shaped distribution. Increasing cigarette prices reduces smokers' willingness to gift high-priced cigarettes but stimulates the consumption of low-price cigarettes as gifts. Pictorial HWLs do not directly influence smokers' intentions to gift cigarettes, but they enhance the effectiveness of price regulation concerning medium-priced cigarette products.

**CONCLUSIONS:**

If the price variance of cigarettes is not reduced, the effect of price regulation will be very limited. Implementing combined interventions of pictorial HWLs and price regulation or modifying the pricing structure of tobacco products may yield stronger control outcomes.

## INTRODUCTION

The direct consumption of tobacco by smokers is considered the core of tobacco control. Existing literature has comprehensively explored various variables such as price regulation, product packaging, and retail outlets. However, there is currently a lack of in-depth research on indirect forms of consumption, such as gift-giving. The gifting of cigarettes may not only increase the recipient’s risk of using more tobacco or reduce their willingness to quit smoking, but also easily lead to the formation of a tobacco gift exchange chain, increasing the likelihood of the gift-givers exposure to tobacco^1^. When tobacco gifts become symbols of social capital and status, it inadvertently legitimizes smoking. Despite the evident public health risks associated with tobacco gifts, they hold a special status as gifts in many cultures^2^. As early as the 15th century, cigarettes began to gain popularity as social gifts in North America and Europe^3^. From the 1930s to the 1960s, cigarettes were a common Christmas gift in the United States^4^. Tobacco is one of the most popular gifts in Indian social relationships, often presented in ceremonies to dignitaries^5^. In Russia, cigarettes serve as a significant social currency and gift, especially among intellectuals^6^.

In traditional Chinese culture, gifts are crucial elements in building and developing social relationships^7^. Since at least the 1980s, some high-price cigarettes have become significant reciprocal gifts^8^. Tobacco companies also leverage cigarette brands and packaging to promote the popularization of cigarette social culture. For Chinese smokers, cigarettes are deemed essential for special occasions and serve as an effective ‘currency’ to facilitate work^9^. Whether for social or business purposes, offering and sharing cigarettes are common practices, considered both polite and a good way to make friends^10^. Approximately 90% of smokers in China either receive or give cigarettes as gifts, and high-price cigarettes are often regarded as symbols of status and respect in social gatherings^11^. Research by Rich and Xiao^12^ found that the practice of gifting or engaging in bribes with cigarettes is common in China. On average, Chinese smokers receive gift cigarettes five times a year^13^.

In regions with a smoking social culture, tobacco control efforts are constrained by cultural norms, resulting in an increase in cigarette consumption among smokers. Research by Wu et al.^14^ has found that social behaviors such as sharing and giving cigarettes can effectively predict smoking patterns. From a sociological perspective, giving cigarettes is a form of social exchange characterized by clear reciprocity^1,2,15^. Givers of gifted cigarettes typically expect to receive an equivalent value of cigarettes in return, leading to an increase in cigarette consumption and overall circulation^16^. Simultaneously, the existence of a cigarette-gifting culture also lends legitimacy to tobacco consumption in the form of cultural norms, influencing the value judgments of smokers, adolescents, quitters, and potential smokers regarding smoking behavior.

In the realm of cigarette social studies, existing studies have fully explained the social dynamics^16^ and cultural drive^12,17^ of Chinese cigarette social behavior, but there are few studies on how to curb cigarette social behavior in Chinese society. Studies in Western countries suggest that the dissemination of public health knowledge can effectively restrain tobacco-related social behaviors. For instance, the highly successful public health education in the United States during the 1960s contributed to cigarettes no longer being perceived as fashionable social gifts^18^. In China, although the existing education methods aimed at tobacco hazards have effectively improved the public’s awareness of tobacco health risks, their impact on tobacco social behavior is relatively weak^19^. People resort to cultural factors to defend cigarette social behaviors and resist educational methods through the cultural values formed by the gift-giving chain of cigarettes^18,20^.

In the realm of tobacco control measures, existing research predominantly explores the impact mechanism of price control on Chinese smokers’ willingness to quit and direct tobacco consumption^21^. Additionally, the current cigarette packaging in China primarily features textual Health Warning Labels (HWL), with the effectiveness of text HWLs being deemed less powerful than pictorial HWLs^22^. In the future, these two measures are likely to become administrative actions for the Chinese government to further implement tobacco control, either independently or in conjunction. However, the potential impact of these measures on cigarette gifting in the Chinese market has not received sufficient attention.

Given the aforementioned conflicts between theory and practice, this study aims to address the following questions through survey experiments: 1) ‘How will price regulation and pictorial HWLs impact smokers’ willingness to gift cigarettes?’, and 2) ‘What effects will the combination of price regulation and pictorial HWLs have on smokers’ willingness to gift cigarettes?’.

## METHODS

### Study design

In this study, we adopted a survey experiment method. The survey-experiment method avoids many endogenous problems of cross-sectional and panel survey data^23^, combines the strengths of survey and experiment, and is also conducive to expanding the sample size. In terms of the types of cigarettes used in the experiment, we selected three cigarettes, which have relatively good sales all year round and can represent the high-, medium- and low-price products of the tobacco industry. Low-priced cigarettes are 10 RMB/pack, medium-priced cigarettes are 20 RMB/pack, and high-priced cigarettes are 45 RMB/pack (1000 Chinese Renminbi about US$140).

The survey was administered to four groups of respondents, each presented with different cigarette prices and external packaging images (Tables 1 and 2). The first group served as the control group, where the survey showcased the packaging and prices of various cigarettes currently available on the market. This group of respondents was used as the baseline to investigate the potential impact of prices and pictorial HWLs on smokers. The second group was the price treatment group, and the survey displayed the packaging of various cigarettes to these participants, but participants were informed in the questionnaire that the prices of all cigarettes had doubled. The third group was the picture HWL treatment group, and the survey presented the prices of various cigarettes on the market to these participants, but all cigarette packages included pictorial HWLs in the questionnaire. The fourth group was the price-picture treatment group, where in this survey, the prices of all cigarettes doubled, and the external packaging of all cigarettes included pictorial HWLs.

**Table 1:** Warning packages and prices applied in survey experiment

*Categories*	*Market prices per carton (RMB)*	*Experiment prices per carton (RMB)*	*Current packages*	*Experiment packages*
Low-priced	100	200	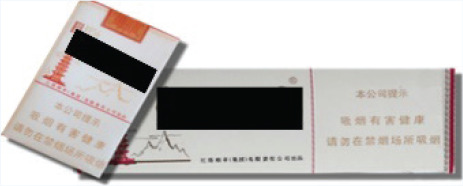	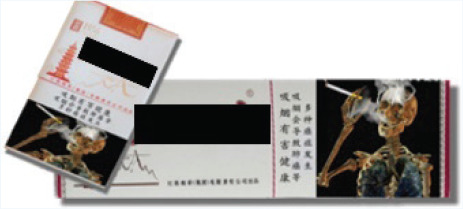
Medium-priced	230	460	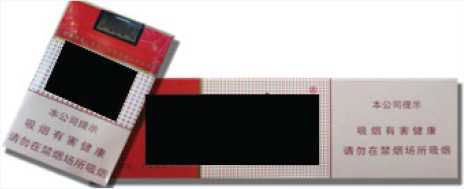	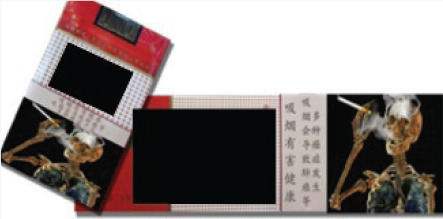
High-priced	450	900	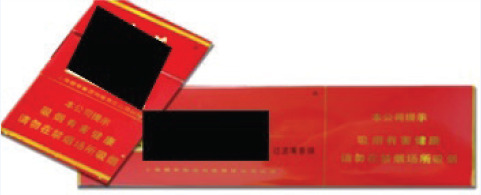	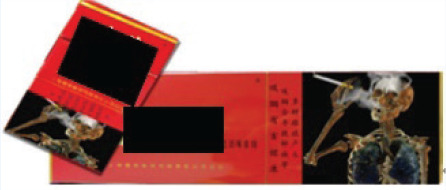

RMB: 1000 Chinese Renminbi about US$140.

**Table 2:** Descriptive statistics

*Characteristics*	*Group 1 Mean (SD)*	*Group 2 Mean (SD)*	*Group 3 Mean (SD)*	*Group 4 Mean (SD)*	*All Mean (SD)*
Low-priced cigarette gifting willingness	1.906 (1.161)	2.115 (1.126)	2.066 (1.197)	2.456 (1.153)	2.186 (1.179)
Medium-priced cigarette gifting willingness	2.780 (1.211)	2.495 (1.207)	2.823 (1.302)	2.614 (1.199)	2.676 (1.235)
High-priced cigarette gifting willingness	3.262 (1.304)	2.985 (1.324)	3.207 (1.300)	2.670 (1.264)	2.981 (1.315)
Age (years)	40.052 (10.797)	38.570 (11.054)	38.974 (10.844)	41.466 (10.476)	39.993 (10.797)
Gender	0.948 (0.223)	0.920 (0.272)	0.930 (0 .256)	0.890 (0.313)	0.917 (0.276)
Education level	4.010 (1.210)	4.350 (1.079)	3.863 (1.268)	3.633 (1.308)	3.901 (1.263)
Income	11.391 (11.518)	13.690 (13.914)	12.048 (16.074)	13.891 (14.681)	12.908 (14.413)
Marital status	0.791 (0.408)	0.690 (0.464)	0.742 (0.439)	0.796 (0.403)	0.760 (0.427)
Parental status	0.754 (0.432)	0.700 (0.459)	0.723 (0.448)	0.788 (0.409)	0.748 (0.434)
Cigarettes per day	1.817 (0.890)	1.755 (0.786)	1.779 (0.814)	2.040 (0.922)	1.875 (0.871)
Average time (hours) to first cigarette after wakening	2.565 (1.098)	2.835 (1.026)	2.664 (1.051)	2.501 (1.028)	2.620 (1.053)
Perceived harm of personal smoking	2.665 (0.854)	2.650 (0.755)	2.720 (0.737)	2.660 (0.782)	2.674 (0.779)
Perceived harm of secondhand smoke	2.576 (0.860)	2.530 (0.801)	2.583 (0.825)	2.544 (0.830)	2.557 (0.828)
**Total**	191	200	271	373	1035

### Sample participants and data collection

This study primarily collected samples from adults aged ≥19 years. Convenience and snowball sampling methods were employed to recruit participants. Undergraduate students from a large research university served as research assistants for data collection. We recruited a total of 80 undergraduate students aged ≥19 years to assist in the distribution of questionnaires. These university students were randomly divided into four groups, and these four groups of students distributed the four sets of questionnaires used in the survey to smokers through their social networks in the community. The total sample size was 1035 participants. Group 1 (control group) involved maintaining the current price and packaging, comprising 191 participants. Group 2 (price experimental group) featured a doubled price while maintaining the current packaging, comprising 200 participants. Group 3 (image experimental group) maintained the current price but introduced a pictorial health warning label (HWL) package, comprising 271 participants. Group 4 (price-image experimental group) incorporated both a doubled price and a pictorial HWL package, comprising 373 participants. The sample sizes of the third and fourth groups were significantly higher than those of the first and second groups, possibly because new cigarette packaging was used in the third and fourth groups, creating a higher novelty and, consequently, a higher response rate from the participants.

Considering the geographical differences within China, this study collected data from various regions across the country. The sample covered 30 provinces and regions (excluding Tibet) and other regions, including Hong Kong and Macau. There were 26 participants from the Northeast, 298 participants from the East, 151 participants from the North, 221 participants from the Central region, 107 participants from the South, 116 participants from the Southwest, 83 participants from the Northwest, and 33 participants from other regions, including Hong Kong and Macau.

This study obtained approval from the university’s academic ethics committee. Prior to data collection, all research assistants were informed about the overall research protocol and electronically signed informed consent forms. To ensure participant anonymity, all identifiable information was removed. Research data were stored on password-protected electronic devices.

### Measurement

The dependent variable is cigarette gifting willingness. Regarding the three selected cigarettes, we asked participants to what extent they were willing to give low-priced, medium-priced, and high-priced cigarettes as gifts to relatives and friends. Here, participants’ willingness to gift cigarettes after receiving the experimental treatment was measured. This question uses a five-point Likert scale: 1=very unwilling, 2=relatively unwilling, 3=average, 4=relatively willing, and 5=very willing.

The survey also collected sociodemographic characteristics of participants, including age, gender, education level, income, marital status, and whether they have children. The age (years) variable is based on the reported birthdate. Gender was a binary variable (1=male, 0=female). The education variable uses a seven-point indicator (1=elementary school, 2=junior high school, 3=high school, 4=college, 5=Bachelor’s degree, 6=Master’s degree, and 7=Doctoral degree). The income variable is the annual income filled in by the participants in units of 1 wan RMB (10000 RMB or about US$1400). Marital status is a binary variable: 0=never married, 1=married/cohabiting/divorced/widowed. Parental status is also a binary variable: 0=no children, 1=have children.

In addition, we also used two other types of variables as control variables for statistical inference analysis. The first category is nicotine dependence, including the average daily usage of cigarettes and the average time to smoke the first cigarette after waking up in the morning. Average daily cigarette consumption was measured using a four-point indicator, 1= ≤10, 2= 11–20, 3= 21–30, 4= ≥31 cigarettes. The other category is smokers’ perceived harm of cigarettes, including the perceived harm of personal smoking and the harm of secondhand smoke.

Two variables regarding daily cigarette consumption price were measured before treatment. Personal consumption price refers to smokers’ price preference for cigarettes that they usually use themselves, in RMB/pack, and gifting cigarette price refers to smokers’ price preference for cigarettes that they usually give as gifts to relatives and friends, in RMB/carton. Because when giving gifts, Chinese people usually like to give a complete cartoon, including ten packs of cigarettes.

### Statistical analysis

All statistical analyses were performed using Stata 15.1. The descriptive statistics section reports the mean and standard deviation. All multivariable relationships were estimated using multiple linear regression models and 95% confidence intervals are reported. All regression models included demographic characteristics, nicotine dependence, and perceived harm of cigarettes as control variables. Four levels of significance were distinguished using p<0.1, p<0.05, p<0.01, and p<0.001. A smaller -value indicates that there is stronger evidence to reject the null hypothesis and accept the alternative hypothesis.

## RESULTS

### Descriptive analysis

Table 3 presents the sub-group means and overall means of the dependent variables. The willingness to gift low-priced cigarettes has a mean of 2.19 (SD=1.18). For medium-priced cigarettes, the willingness to gift has a mean of 2.68 (SD=1.24). High-priced cigarettes exhibit a willingness to gift

**Table 3:** A survey experiment of the impact of low-priced cigarette prices and pictorial HWLs on cigarette gifting willingness, China, 2023 (N=1035)

*Variables*	*Model 1 (Group 1 vs 2)*		*Model 2 (Group 1 vs 3)*		*Model 3 (Group 1 vs 4)*	
	*β (95% CI)*	*p*	*β (95% CI)*	*p*	*β (95% CI)*	*p*
**Group indicator**	0.26* (0.03–0.50)	0.03	0.16 (-0.06–0.39)	0.15	0.49*** (0.28–0.69)	0.000
**Control variable**						
Age (years)	0.00 (-0.01– -018)	0.68	0.01 (-0.01–0.02)	0.36	-0.01 (-0.02–0.00)	0.14
Gender	-0.39^†^ (-0.85–0.07)	0.10	-0.25 (-0.71–0.22)	0.29	-0.74*** (-1.08 – -0.40)	0.000
Education level	-0.05 (-0.16–0.06)	0.40	0.04 (-0.06–0.13)	0.46	-0.02 (-0.10–0.06)	0.69
Income	-0.01^†^ (-.018–0.00)	0.06	-0.00 (-0.01–0.01)	0.96	-0.00 (-0.02–0.00)	0.83
Marital status	-0.13 (-0.57–0.31)	0.55	-0.40* (-0.80 – -0.00)	0.05	-0.19 (-0.14–0.51)	0.26
Children	0.15 (-0.32–0.61)	0.54	0.35 (-0.08–0.78)	0.11	0.19 (-0.16–0.54)	0.29
Cigarettes per day	-0.08 (-0.23–0.08)	0.34	-0.07 (-0.22–0.08)	0.38	0.06 (-0.20–0.00)	0.35
Average time to first cigarette after wakening	-0.13* (-0.25 – -0.01)	0.04	-0.10 (-0.21–0.02)	0.11	-0.10^†^ (-0.20–0.00)	0.06
Perceived harm of personal smoking	0.10 (-0.09 – 0.29)	0.29	0.06 (-0.12 – 0.23)	0.52	0.12 (-0.04–0.27)	0.14
Perceived harm of secondhand smoke	-0.06 (-0.24 – 0.13)	0.55	0.04 (0.88–2.98)	0.71	-0.10 (-0.24–0.05)	0.18
Adjusted R^2^	0.02		0.00		0.09	
**Total**	391		462		564	

† p<0.1,

* p<0.05,

** p<0.01,

*** p<0.001.

with a mean of 2.981 (SD=1.32). The control variables depict the sample characteristics: the age variable has a mean of 39.99 (SD=10.80), the gender variable has a mean of 0.92, indicating that 92% of the participants are male. The education level variable has a mean of 3.90 (SD=1.26). The income variable has a mean of 12.91 (SD=14.41). The marital status variable has a mean of 0.76 (SD=0.43), and the parental status variable has a mean of 0.75 (SD=0.43). This suggests that, on average, participants are aged around 40 years, 92% are male, the average education level is slightly below a Bachelor’s degree, the average income is around 130000 RMB, 76% of participants are in a married, divorced, cohabitating, or widowed status, and 75% of participants have children.

In the nicotine dependence category, the overall daily cigarette consumption has a mean of 1.88 (SD=0.87), and the average time to the first cigarette after waking is 2.62 hours (SD=1.05). For the perceived health risk category, the overall perceived personal risk of smoking has a mean of 2.67 (SD=0.78). The perceived risk of secondhand smoke has an overall mean of 2.56 (SD=0.83).

**Figure 1:**
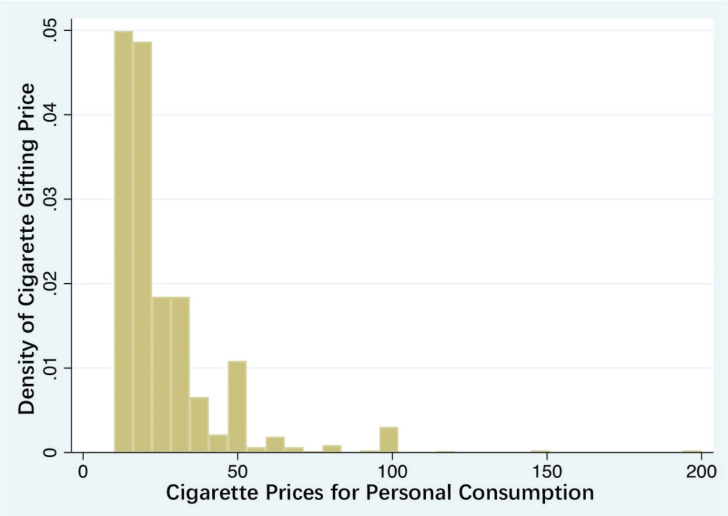
The distribution of cigarette prices for personal consumption

**Figure 2:**
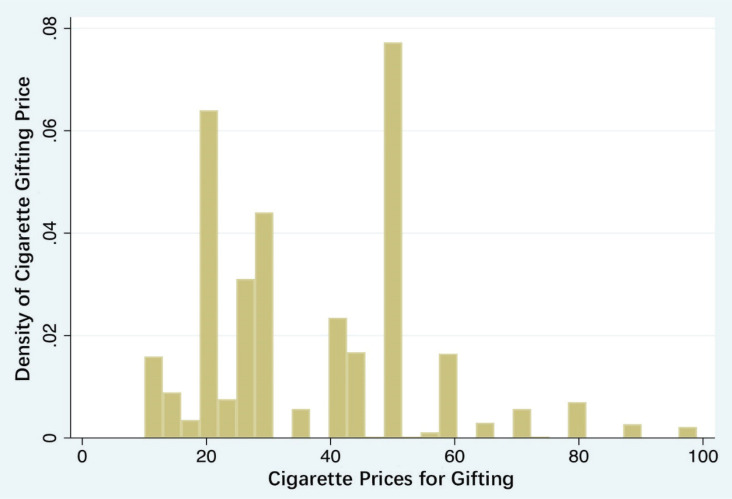
The distribution of cigarette price for social gifts consumption

Figure 1 reports the density of personal cigarette consumption prices, indicating that low-priced cigarettes in the range of 10–20 RMB are the most popular, presenting an unimodal distribution. Figure 2 reports the density of gifted cigarette prices, showing that low-priced cigarettes in the range of 10–20 RMB are the most popular, forming an L-shaped distribution. Peaks appear around 20, 50, and 100 RMB, with the highest consumption of cigarette gifts occurring around 50 RMB, demonstrating a W-shaped distribution.

### Multivariate analysis

Tables 3–5 present the estimates of the multivariate analysis. Each table compares the estimates of Models 1–3. Model 1 is based on the subsample of the control group (Group 1) and the price treatment group (Group 2), comprising a total of 391 participants. Model 2 is based on the subsample of the control group (Group 1) and the pictorial HWLs treatment group (Group 3), with a total of 462 participants. Model 3 is based on the subsample of the control group and the price–pictorial HWLs treatment group, with a total of 564 participants.

**Table 4:** A survey experiment of the impact of medium-priced cigarette prices and pictorial HWLs on cigarette gifting willingness, China, 2023 (N=1035)

*Variables*	*Model 1 (Group 1 vs 2)*		*Model 2 (Group 1 vs 3)*		*Model 3 (Group 1 vs 4)*	
	*β (95% CI)*	*p*	*β (95% CI)*	*p*	*β (95% CI)*	*p*
**Treatment**						
Group indicator	-0.20 (-0.45–0.05)	0.11	0.05 (-0.18–0.29)	0.66	-0.20^†^ (-0.41–0.02)	0.07
**Control variable**						
Age (years)	0.003 (-0.01–0.02)	0.72	0.01 (-0.01–0.02)	0.29	-0.01* (-0.03 – -0.00)	0.03
Gender	-0.30 (-0.78–0.19)	0.23	0.17 (-0.32–0.66)	0.51	-0.36^†^ (-0.72–0.00)	0.05
Education level	-0.11^†^ (-0.23–0.00)	0.05	-0.01 (-0.11–0.09)	0.88	0.02 (-0.07–0.10)	0.69
Income	-0.01 (-0.02–0.00)	0.15	-0.00 (-0.01–0.01)	0.41	0.00 (-0.01–0.01)	0.78
Marital status	0.11 (-0.36–0.57)	0.65	-0.26 (-0.68–0.17)	0.24	0.10 (-0.25–0.44)	0.59
Children	-0.07 (-0.56–0.43)	0.80	0.27 (-0.19–0.72)	0.24	0.21 (-0.16–0.58)	0.26
Cigarettes per day	-0.07 (-0.24–0.09)	0.38	-0.03 (-0.19–0.13)	0.71	0.09 (-0.04–0.21)	0.16
Average time to first cigarette after wakening	-0.12^†^ (-0.25–0.01)	0.06	-0.14* (-0.26–0.02)	0.03	-0.09 (-0.19–0.02)	0.12
Perceived harm of personal smoking	0.10 (-0.10–0.30)	0.35	0.21* (0.03–0.40)	0.02	0.12 (-0.04–0.27)	0.15
Perceived harm of secondhand smoke	-0.09 (-0.29–0.11)	0.36	0.02 (-0.16–0.19)	0.86	-0.10 (-0.25–0.05)	0.19
Adjusted R^2^	0.03		0.02		0.02	
**Total**	391		462		564	

† p<0.1,

* p<0.05,

** p<0.01,

*** p<0.001.

**Table 5:** A survey experiment of the impact of high-priced cigarette prices and pictorial HWLs on cigarette gifting willingness, China, 2023 (N=1035)

*Variables*	*Model 1 (Group 1 vs 2)*		*Model 2 (Group 1 vs 3)*		*Model 3 (Group 1 vs 4)*	
	*β (95% CI)*	*p*	*β (95% CI)*	*p*	*β (95% CI)*	*p*
**Treatment**						
Group indicator	-0.40** (-0.66 – -0.14)	0.00	-0.08 (-0.32–0.16)	0.49	-0.54*** (-0.77 – -0.32)	0.000
**Control variable**						
Age (years)	-0.00 (-0.02–0.01)	0.63	-0.01 (-0.02–0.01)	0.34	-0.02** (-0.03 – -0.01)	0.00
Gender	0.04 (-0.48–0.56)	0.88	0.34 (-0.16–0.84)	0.18	-0.06 (-0.44–0.32)	0.76
Education level	0.09 (-0.03–0.22)	0.13	0.02 (-0.08–0.13)	0.67	0.12** (0.03–0.21)	0.01
Income	0.01* (0.00–0.02)	0.03	0.02*** (0.01–0.02)	0.00	0.01** (0.00–0.02)	0.01
Marital status	0.04 (-0.46–0.53)	0.89	-0.17 (-0.60–0.26)	0.43	0.26 (-0.10–0.62)	0.16
Children	-0.48^†^ (-1.00–0.05)	0.08	-0.21 (-0.67–0.26)	0.38	-0.12 (-0.50–0.27)	0.55
Cigarettes per day	0.01 (-0.17–0.18)	0.93	-0.02 (-0.18–0.14)	0.78	-0.04 (-0.17–0.09)	0.52
Average time to first cigarette after wakening	0.12^†^ (-0.02–0.25)	0.09	-0.01 (-0.14–0.12)	0.88	0.02 (-0.09–0.13)	0.67
Perceived harm of personal smoking	-0.02 (-0.24–0.19)	0.82	0.12 (-0.07–0.31)	0.22	0.11 (-0.06–0.27)	0.19
Perceived harm of secondhand smoke	-0.19 (-0.40–0.02)	0.07	-0.13 (-0.31–0.05)	0.17	-0.23** (-0.38 – -0.07)	0.01
Adjusted R^2^	0.06		0.04		0.10	
**Total**	391		462		564	

† p<0.1,

* p<0.05,

** p<0.01,

*** p<0.001.

Table 3 presents the estimated results of participants’ willingness to gift low-priced cigarettes. Model 1 indicates a significant increase in smokers’ willingness to gift low-priced cigarettes after being exposed to the treatment of increased cigarette prices (β= -0.26; 95% CI: -0.03–0.50, p<0.05). Model 2 suggests that adding pictorial HWLs to cigarette packs did not lead to a significant change in smokers’ willingness to gift low-priced cigarettes. Model 3 indicates that when the price increase is combined with pictorial HWLs, smokers’ willingness to gift low-priced cigarettes remains significantly increased (β=0.49; 95% CI: 0.28–0.69, p<0.001).

Table 4 presents the estimated results of smokers’ willingness to gift medium-priced cigarettes. Models 1 and 2 indicate that neither the price increase nor the addition of pictorial HWLs significantly affect participants’ willingness to gift medium-priced cigarettes. However, Model 3 suggests that when the price increase is combined with pictorial HWLs, smokers’ willingness to gift medium-priced cigarettes significantly decreases (β= -0.20; 95% CI: -0.41–0.02, p<0.1).

Table 5 presents the estimated results of smokers’ willingness to gift high-priced cigarettes. Model 1 indicates that a cigarette price increase significantly decreases smokers’ willingness to gift high-priced cigarettes (β= -0.40; 95% CI: -0.66 – -0.14, p<0.01). Model 2 suggests that the addition of pictorial HWLs does not significantly affect participants’ willingness to gift high-priced cigarettes. However, Model 3 demonstrates that when the price increase is combined with graphic health warning labels, smokers’ willingness to gift high-priced cigarettes significantly decreases (β= -0.54; 95% CI: -0.77 – -0.32, p <0.001).

Regarding the control variables, the results in Table 3 indicate that female gender (β_model1_=0.39; 95% CI: -0.85–0.07, β_model3_= -0.74; 95% CI: -1.08 – -0.40), lower income levels (β= -0.01; 95% CI: -0.018–0.00), unmarried status (β_model2_= -0.40; 95% CI: -0.80 – -0.00), and lower nicotine dependence (waiting time variable, β_model1_= -0.13; 95% CI: -0.25 – -0.01, β_model3_= -0.10; 95% CI: -0.20–0.00) are associated with a greater willingness among participants to gift lowpriced cigarettes. The results in Table 4 indicate that participants who are younger (β_model3_= -0.01; 95% CI: -0.03 – -0.00), female (β_model3_= -0.36; 95% CI: -0.72– 0.00), have lower education level (β_model1_=0.11; 95% CI: -0.23–0.00), exhibit lower nicotine dependence (waiting time variable, β_model1_= -0.12; 95% CI: -0.25– 0.01, β_model2_= -0.14; 95% CI: -0.26–0.02), and perceive higher health risks (harm of personal smoking variable, β_model2_=0.21; 95% CI: 0.03–0.40) may be more willing to gift medium-priced cigarettes. The results in Table 5 suggest that participants who are younger (β_model3_= -0.02; 95% CI: -0.03 – -0.01), have a higher level of education β_model3_=0.12; 95% CI: 0.03–0.21), higher income (β_model1_=0.01; 95% CI: -0.00–0.02, β_model2_=0.02; 95% CI: 0.01–0.02, β_model3_=0.01; 95% CI: 0.00–0.02), do not have children (β_model1_= -0.48; 95% CI: -1.00–0.05), exhibit higher nicotine dependence (waiting time variable, β_model1_=0.12; 95% CI: -0.02–0.25), and perceive lower health risks (harm of second-hand smoke variable, β_model3_= -0.23; 95% CI: -0.38 – -0.07) may be more willing to gift high-priced cigarettes.

## DISCUSSION

This study examines the effects of cigarette price regulation and pictorial HWLs on cigarette packaging on the willingness to gift cigarettes through a randomized survey experiment. The results of multivariate analysis show that the impact of price regulation on cigarettes of different prices is not consistent. In the extreme case of doubling the price, it only reduces the participants’ willingness to gift high-priced cigarettes, and enhances the participants’ willingness to gift low-priced cigarettes. It can be seen that the increase in the price of cigarettes did not cause smokers to give up choosing cigarettes as gifts, but tended to choose lower grade alternatives. While adding pictorial HWLs alone may not effectively decrease cigarette gifting willingness, the combined effect of pictorial warnings and price regulation significantly reduces participants’ willingness to use medium-priced cigarettes as substitutes for high-priced ones.

Although price regulation is the most important means of tobacco control for most governments, the price variance of cigarettes in the Chinese market is far greater than that in European and American countries. Therefore, when the price of cigarettes rises, smokers tend to be more inclined to look for alternatives of different brands rather than give up cigarette gifting^23,24^. This study finds a similar substitution pattern in cigarette gifting. Moreover, since the main price range of gift cigarettes is significantly higher than the consumption price range of smokers’ personal consumption cigarettes, even under the extreme price regulation scenario set in this work, there is still room for alternative consumption. While the conclusions of this study may not be directly applicable to the real world, the findings of this work are thought-provoking. The measure of incorporating pictorial Health Warning Labels (HWLs) on cigarette external packaging has been widely adopted by numerous countries, and in the future, it is highly likely to be implemented by the Chinese government. China has also joined the World Health Organization Framework Convention on Tobacco Control (FCTC), which categorizes measures to reduce tobacco demand into nine recommendations, including pricing and tax measures as well as non-price measures^25^. Without changing the price variance, it is hard to believe that a gentle price regulation in practice can have a significant impact on cigarette gift consumption. On the other hand, considering the particularity of gift consumption, even if the overall price of tobacco rises, the high variance of price differences will drive gift-givers to continue to consider gifting cigarettes, because cigarettes can still achieve actors’ motivations, including accumulating social capital, demonstrating social status and economic strength, or maintaining social relationships^5,26,27^. Therefore, a simple tax increase measure alone may not achieve significant results, and changing the price structure of tobacco may achieve a stronger containment effect.

In addition, just adding pictorial HWLs on the outer packaging of cigarette packs does not have a significant impact on smokers’ willingness to consume gift cigarettes. Existing studies have shown that pictorial HWLs can help consumers recognize more effectively the health risks of smoking than from text warning packaging, reduce the willingness to consume gift cigarettes, and prompt them to quit smoking early^28^. However, this study shows that simply adding pictorial HWLs cannot prompt smokers to give up choosing cigarettes as a social gift. But pictorial HWLs can strengthen the curb effect of price regulation on medium-priced cigarettes. Since medium-priced cigarettes are one of the most important consumer goods for gift cigarette consumption and cannot be controlled simply by raising prices, the superimposed intervention of pictorial HWLs and price regulation may be the only known control method. Therefore, the intervention effect of pictorial HWLs on cigarette gifting still cannot be ignored, and further exploration is still needed.

### Limitations

This study has some limitations. First, this study sets an extreme price regulation scenario in the questionnaire, which may limit the generalization of the conclusions to real price regulation. Second, this study adopted a snowball sampling method, which may lead to insufficient representative samples. Third, as the data utilized in this study are self-reported survey responses, there may be the presence of social desirability effects and other measurement errors. Fourth, given the higher response rates observed in the third and fourth groups, potential response bias cannot be overlooked. Fifth, since we utilized non-time-series data, further investigation is required to understand specific temporal variations. Sixth, the findings of this study may have applicability in regions where the culture of cigarette gifting and social interactions prevails. However, it may not be directly applicable in Western countries such as Europe and the United States.

## CONCLUSIONS

This study uses a randomized survey experiment to examine the impact of two tobacco control measures that the Chinese government may adopt in the future on cigarette gifting: price regulation and pictorial HWLs on cigarette packaging. Raising the price of cigarettes reduces smokers’ willingness to consume high-priced cigarettes but promotes gifting willingness of low-priced cigarettes. Under the existing price variance, the alternative products of gift cigarettes leads to a small price elasticity, so the impact of raising cigarette prices on cigarette gifting is relatively limited. Simply adding pictorial HWLs to cigarette packages cannot affect smokers’ willingness to consume gift cigarettes, either. However, under the superimposed effect of price regulation, pictorial HWLs can strengthen the curb effect of price regulation on medium-priced cigarettes.

## Data Availability

The data supporting this research are available from the authors on reasonable request.
